# The Analyses of Cetacean Virus-Responsive Genes Reveal Evolutionary Marks in Mucosal Immunity-Associated Genes

**DOI:** 10.1007/s10528-022-10221-8

**Published:** 2022-03-25

**Authors:** Oksung Chung, Ye-Eun Jung, Kyeong Won Lee, Young Jun An, Jungeun Kim, Yoo-Rim Roh, Jong Bhak, Kiejung Park, Jessica A. Weber, Jaehun Cheong, Sun-Shin Cha, Jung-Hyun Lee, Hyung-Soon Yim

**Affiliations:** 1 Clinomics, Ulsan, 44919 Republic of Korea; 2 Department of Chemistry and Nanoscience, Ewha Womans University, Seoul, 03760 Republic of Korea; 3 Marine Biotechnology Research Center, Korea Institute of Ocean Science and Technology, 385 Haeyang-ro, Busan, 49111 Republic of Korea; 4 Personal Genomics Institute, Genome Research Foundation, Cheongju, 28160 Republic of Korea; 5 Department of Marine Biotechnology, Korea University of Science and Technology, Daejeon, 306-350 Republic of Korea; 6 Department of Biomedical Engineering, School of Life Sciences, Ulsan National Institute of Science and Technology (UNIST), Ulsan, 44919 Republic of Korea; 7 Sangmyung University, Cheonan, 31066 Republic of Korea; 8 Department of Biology, University of New Mexico, Albuquerque, NM 87131 USA; 9 Department of Molecular Biology, Pusan National University, Busan, 46241 Republic of Korea

**Keywords:** Cetacean, Response to virus, PSG, Mucosal immunity, Interferon ε

## Abstract

Viruses are the most common and abundant organisms in the marine environment. To better understand how cetaceans have adapted to this virus-rich environment, we compared cetacean virus-responsive genes to those from terrestrial mammals. We identified virus-responsive gene sequences in seven species of cetaceans, which we compared with orthologous sequences in seven terrestrial mammals. As a result of evolution analysis using the branch model and the branch-site model, 21 genes were selected using at least one model. IFN-ε, an antiviral cytokine expressed at mucous membranes, and its receptor IFNAR1 contain cetacean-specific amino acid substitutions that might change the interaction between the two proteins and lead to regulation of the immune system against viruses. Cetacean-specific amino acid substitutions in IL-6, IL-27, and the signal transducer and activator of transcription (STAT)1 are also predicted to alter the mucosal immune response of cetaceans. Since mucosal membranes are the first line of defense against the external environment and are involved in immune tolerance, our analysis of cetacean virus-responsive genes suggests that genes with cetacean-specific mutations in mucosal immunity-related genes play an important role in the protection and/or regulation of immune responses against viruses.

**Supplementary Information:**

The online version contains supplementary material available at 10.1007/s10528-022-10221-8.

## Introduction

Cetaceans are special mammals that evolved to return from terrestrial to aquatic habitats and diverged from their land-dwelling ancestors. To survive in aquatic environments as mammals, cetaceans underwent physiological and morphological adaptation, for which the molecular bases have been suggested by recent genome analysis (Yim et al. 2014). From cetacean-specific genome information, many genes in cetaceans have been identified to cope with environmental stresses, such as high salt concentration, hypoxia, low temperature, and deep diving. However, physiological protective systems against infectious microorganisms have not yet been investigated at the genome level.

Viruses are the most abundant biological beings and its amount is estimated to be 10^30^ in ocean. The abundance of viruses is approximately 15 times larger than that of archaea and bacteria (Fuhrman 1999; Suttle 2007). Marine viruses play major roles in biodiversity, population dynamics, and biogeochemical cycling (Suttle 2005). In addition to these ecological roles, infectious viruses are threats to the survival of cetacean populations. A variety of viruses have been detected in cetaceans (Van Bressem et al. 1999), and severe lesions have been reported due to viral infection. Cetacean viruses drew attention after the advent of morbillivirus mortalities in Phocoena along the coasts of Ireland, England, and the Netherlands in the northeastern Atlantic from 1988 to 1990 (Kennedy et al. 1988) and a lethal epizootic of morbillivirus in *Stenella coeruleoalba* in the Mediterranean from 1990 to 1992 (Domingo et al. 1990). Cetacean defense systems against viruses should be required for protection from repetitive exposure to viruses. Due to epidemics with high mortality, cetacean morbilliviruses (CeMV) have been more carefully monitored and their phylogeny and pathogenesis have been investigated (Van Bressem et al. 2014). The signaling lymphocyte activation molecule (SLAM, CD150) in immune cells has been described as a cellular receptor for measles virus, a highly contagious human morbillivirus (Tatsuo et al. 2000). Although variations in amino acids in the variable region of SLAM in cetaceans were examined to investigate the susceptibility of cetaceans to CeMV (Shimizu et al. 2013) and single-nucleotide polymorphisms (SNPs) of immunity-related genes in *S. coeruleoalba* and *P. phocoena* were previously identified as a potential tool for studying the immunogenome (Stejskalova et al. 2017), the molecular machinery that underlies the cetacean antiviral system has not been investigated at the genomic scale.

Herein, we present the gene sequences that are clustered to respond to a virus (GO: 0009615 in AmiGO 2) in seven species of cetaceans and compare them to the orthologous sequences of terrestrial mammals. This GO term includes genes corresponding to all processes that result in a change in the state or activity of a cell or organism as a result of viral stimulation. Based on the evolutionary analysis, we suggest that the environment-adapted mucosal immunity-related genes of cetaceans may contribute to the overall immune system against viral insult.

## Materials and Methods

### Virus-Responsive Genes in Cetaceans

The gene information related to the ‘response to virus’ gene ontology (GO) term (GO: 0009615) was downloaded from Amigo (http://amigo.geneontology.org/amigo) (Carbon et al. 2008) and a total of 308 gene lists were obtained (on the 15th of March 2018). ‘Response to virus’ includes GO terms related to viruses, such as defense response to virus and detection of virus. Information of human genes that belong to GO: 0009615 (308 genes) were acquired from the UniGene database and orthologous sequences of 12 mammals (except the bowhead whale) with RefSeq assembly accession numbers were obtained from the NCBI (on the 20th of February 2018). The genome sequence of the Bowhead whale was acquired at The Bowhead Whale Genome Resource (http://www.bowhead-whale.org) (Keane et al. 2015). The coding regions of these genes from bowhead whales were predicted from genomic scaffolds using Exonerate v2.2.0 (Slater and Birney 2005) with ‘-model protein2genome-percent 30’ options. To search for 308 orthologous genes for 13 mammals, human genes were used as a query for blastp with default options and the orthologous genes were found using Reciprocal Best Hit methods. The GenBank accession numbers of all genes used in our study are listed in Table S1. All the genes used in this study were intact and did not include frame shift mutations or premature stop codons.

### Molecular Evolutionary and Comparative Analyses of Virus-Responsive Genes

We performed two sequence alignment algorithms for evolutionary analysis: PRANK program (Löytynoja and Goldman 2005) to align sequences using the codon option and the GUIDANCE tool to perform multiple sequence alignment and filtering using the mafft and codon options (Sela et al. 2015). To estimate the rates of synonymous (*d*_*S*_) and nonsynonymous substitutions (*d*_*N*_), we used the CODEML program of PAML 4.5 (Yang 2007) and the previously reported phylogenetic topology (Nam et al. 2017). The branch model was used to calculate the *d*_*N*_/*d*_*S*_ ratios for cetaceans and the other mammals. Briefly, the one-ratio model was first calculated to estimate the general selective pressure acting on all species with a single *d*_*N*_/*d*_*S*_ ratio for all branches. Next, the two-ratio model was used to separately estimate *d*_*N*_/*d*_*S*_ ratios for cetaceans and other mammals and to calculate their likelihood. The likelihood ratio test (LRT, *P* ≤ 0.05) was used to assess the statistical significance of *d*_*N*_/*d*_*S*_ ratios between cetaceans and other mammals. We identified the rapidly evolving genes (REGs) whose *d*_*N*_/*d*_*S*_ values of the foreground branch were significantly higher than those of the background branch (*P*-value > 0.05). We also used the branch-site model to further test for signatures of positively selected genes (PSGs). The ancestral cetacean branch was defined as foreground branches and the remaining branches were defined as background branches. For each of the foreground and background branches, we have calculated only those that contain at least three species.

### Prediction of the Functional Effects of Amino Acid Changes

For each virus-responsive gene set, multiple sequence alignments of each protein sequence were performed using the Clustal Omega (Sievers et al. 2011) program. These multiple sequence alignments were used to identify cetacean-specific amino acid substitutions. The PolyPhen-2 (Adzhubei et al. 2013) and PROVEAN (Choi and Chan 2015) programs with human protein sequences as queries were used to predict if amino acid substitutions were likely to affect the biological function of the protein. After replacing the cetacean-specific amino acids with the corresponding sites of the human protein sequence, we predicted the possible impact of amino acid substitutions on the structure and function of human proteins. The predictions ‘possibly damaging’ or ‘probably damaging’ from PolyPhen-2 were considered as function-altering replacements, as were the ‘deleterious’ results from PROVEAN. In addition, we predict the effect of amino acid mutations on protein stability using MUpro1.1 (Cheng et al. 2006).

### Construction of Protein–Protein Interaction (PPI) Network

A PPI network was constructed using the Search Tool for the Retrieval of Interacting Genes (STRING) database (https://string-db.org/) (Szklarczyk et al. 2014). Subsequently, the PPI network was visualized by Cytoscape software (Shannon et al. 2003).

## Results

### Evolutionary Analysis of Cetacean Virus-Responsive Genes

To infer the genetic associations that have allowed cetaceans to inhabit virus-rich marine environments, we compared and analyzed virus-responsive genes in cetacean mammals with those from terrestrial mammals. We obtained 308 human virus-responsive genes within the gene ontology of GO: 0009615 and then compared them with orthologous genes in 13 mammal species, including mouse, cat, horse, pig, cattle, elephant, minke whale, beluga whale, killer whale, baiji, sperm whale, bottlenose dolphin, and bowhead whale (Table S1).

When performing alignment to detect molecular evolution, we will inevitably obtain false positives and false negatives (Jordan and Goldman 2011). Errors in these alignments are a major source of false detection when identifying adaptive evolution using CODEML program. However, filtering the alignment can not only reduce false positives but may also lose interesting results. Therefore, we conducted two simulation studies comparing the results produced by filtered and unfiltered alignments for this study.

Using the branch model, we identified 18 genes and 16 genes as REGs between cetaceans and terrestrial mammals in unfiltered and filtered alignments, respectively (Table S2 and S3). Eleven of these genes were detected as REGs in both alignments. However, seven genes (*BST2*, *BTBD17*, *DDX1*, *EIF2AK2*, *IL33*, *OASL*, and *TRAF3*) were found to be REGs only in unfiltered alignments and five genes (*BIRC3*, *BPIFA1*, *GTF2F1*, *IL-27,* and *RRP1B*) were REGs only in filtered alignments.

**Fig. 1:**
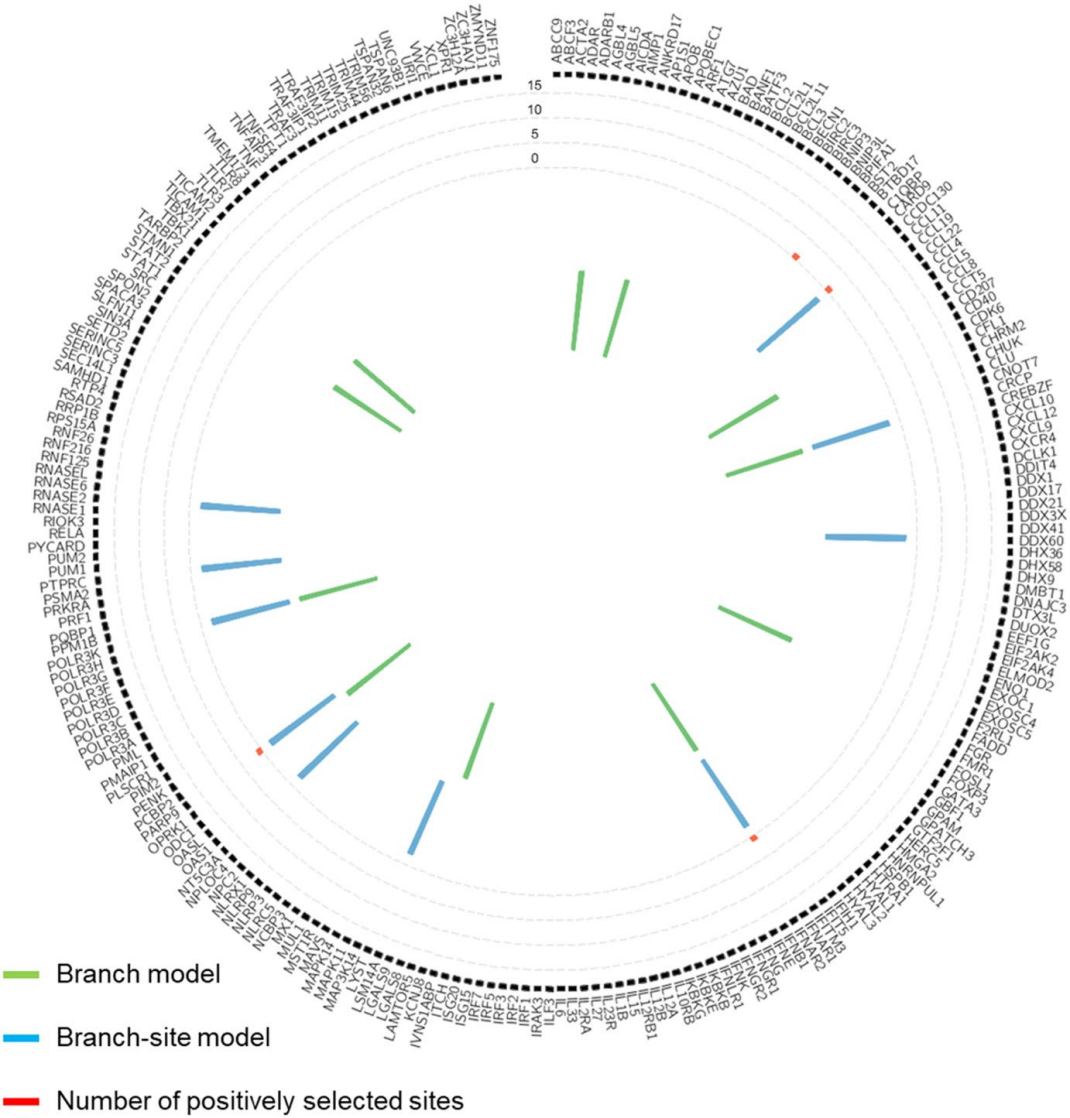
Positively selected genes in cetacean virus-responsive genes. The bars in the four inner circles indicate which of the alternate models (listed in the lower right corner) are most likely. The bars closest to the gene names indicate the number of positively selected sites (posterior probabilities > 0.95) under Bayesian Empirical Bays’ analysis

We also used a branch-site model to detect positive selection in individual codons in the lineage of cetaceans across the phylogeny of all Mammalia. Using the branch-site models, we identified 12 genes and 13 genes that were PSGs of ancestral cetaceans in unfiltered and filtered alignments, respectively (Tables S4 and S5). Ten of these genes were identified commonly as PSGs. Two genes (*HERC5* and *PENK*) were detected as PSGs only using unfiltered alignments and three genes (*ABCC9*, *BPIFA1*, and *OAS1*) were detected as PSGs only using filtered alignments. A total of 21 genes detected in both models using the filtered and unfiltered alignments were identified as PSGs or REGs (Fig. 1), and *CRCP, POLR3K*, and *IFNAR1* were selected as PSGs and REGs simultaneously.

### Nonsynonymous Amino Acid Substitutions Likely Gave Selective Advantages

If only a few amino acids affect important genes involved in evolution, these genes may not be identified as PSGs or REGs (Yang and Bielawski 2000). Therefore, to further investigate genes that might affect cetacean adaptation to the aquatic environment, we also examined the function-altering amino acid substitutions. By aligning the genes from the human and thirteen mammalian genomes, we investigated amino acids that were specifically changed only in the cetaceans. We identified 426 cetacean uAASs (unique amino acid substitutions) from 158 proteins (Table S6) and MUpro1.1 predicted that only 18 sites among all cetacean uAASs increase stability of protein. Most of cetacean uAASs (95.77%) were expected to reduce protein stability. The programs PolyPhen-2 and PROVEAN were used to estimate the functional effects of these unique substitutions. In total, 181 (42.5%) amino acids were predicted as “possibly or probably damaging” by PoPhen-2 and 187 (43.9%) amino acids as “deleterious” by PROVEAN. Of the 308 ‘response to virus’ GO genes, 58 proteins contained function-altering uAAS specific to cetaceans as predicted by both programs. Mucosal immunity-related genes, such as *IFN-ε, IL-6*, *IL-27*, and *IFNAR1*, especially possessed function-altering cetacean uAAS.

### Modular Organization of Virus-Responsive Genes that Evolved Specifically in Cetaceans

**Fig. 2:**
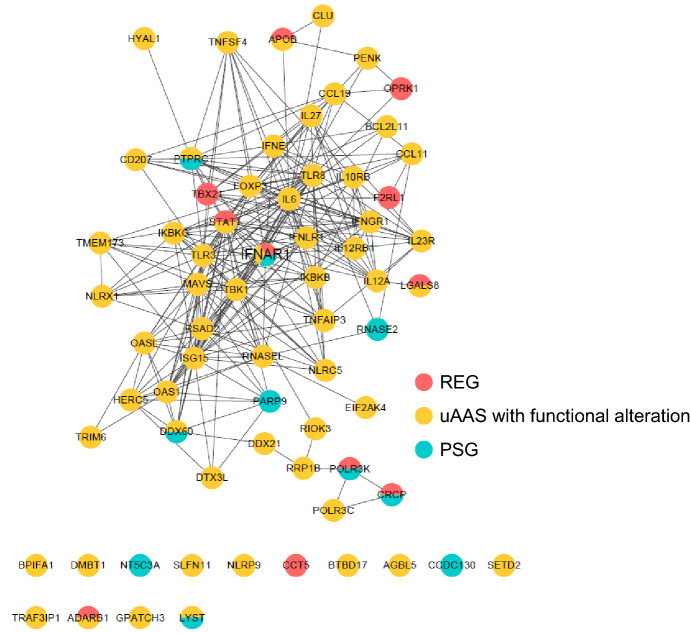
Protein–protein interactions of cetacean genes that were listed by comparative genome analysis The nodes and edges of the network represent cetacean-specific genes and functional associations among these genes, respectively, predicted by STRING database and visualized by Cytoscape. REGs, PSGs, and uAAS with functional alteration are shown in red, cyan, and yellow circle, respectively

To further examine how these cetacean-specific evolving genes participate in antiviral defense, the protein–protein interactions (PPI) of 68 genes (Fig. 2) were predicted using the STRING database (https://string-db.org/) (Szklarczyk et al. 2014) and visualized by Cytoscape (Shannon et al. 2003). The genes in the PPI analysis included REGs, PEGs, and genes with function-altering uAAS. The PPI network was constructed with gene nodes and edges that represented functional associations. Among the genes intertwined in network, three cytokines (IFN-ε, IL-6, and IL-27) are known to be associated with mucosa immune responses. A mucous membrane or mucosa consists of epithelial cell layer(s) and functions as the primary barrier between the external surroundings and the interior of the body. IFN-ε has a unique mucosal constitutive expression profile and antiviral activity (Hardy et al. 2004; Peng et al. 2007). In addition, IL-6 is known to contribute to mucosal immunity (Robinson et al. 2018; Ramsay et al. 1994). IL-27 is a key coordinator of immune regulation in the respiratory tract (Branchett and Lloyd 2019) and is suggested as a potential rescue therapy for acute severe colitis by attenuation of colonic mucosal innate immune response (McLean et al. 2017). In addition, IFNAR1, receptor for type I IFN, including IFN-ε (Harris et al. 2018) and IFN-mediated gene (PARP9), were incorporated in the PSG protein interaction network (Fig. 2). It is likely that IFN-mediated genes in cetacean mucosa may take part in the viral defense system. Taken together, these analyses suggest that cetacean-specific evolved cytokines related to mucosal immunity and IFN-mediated genes may regulate immune response against ocean viruses.

### Functional Modeling of Cetacean-Specific Amino Acid Substitutions

**Fig. 3:**
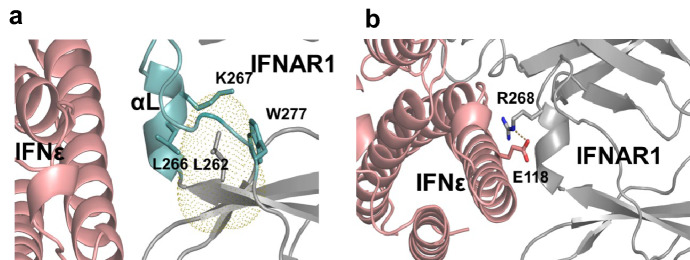
The interface between IFNε (pink) and IFNAR1 (gray). **a** Yellow dots represent the 200% van der Waals radii of Leu262 and αL indicates the ligand-contacting helix. Residues involved in hydrophobic interactions are shown in sticks. The loop propped by Leu262 is highlighted in cyan. **b** A dotted line indicates a salt bridge between Arg268 and Glu118 that are shown in sticks

*IFNAR1* is one of the genes detected as REG and PSG in cetaceans and has function-altering uAAS. In addition, IFNAR1 is one of the representative hub proteins in the PPI analysis (Fig. 2). Compared with human IFNAR1 (hIFNAR1), cetacean IFNAR1 (cIFNAR1) has three substitutions (L262S, N285D, and F503S). The positively selected Ser-262 in the *cIFNAR1* gene (Bayes Empirical Bayes analysis 0.952) was occupied in the fibronectin type 3 (FN3) domain and predicted to have a function-altering effect (Table S6). The L262S replacement of cIFNAR1 is located in the ligand-binding interface, whereas the positions of two other replacements have no relation with the ligand binding. In the structure of hIFNAR1 (PDB code: 3SE4), Leu262 props the structure of a loop (residues 263–277) containing a ligand-contacting helix via tight hydrophobic contacts with Leu266, Trp277, and the aliphatic portion of the Lys267 side chain (Fig. 3a). The three hydrophobic residues are conserved in cIFNAR1 and it is thus likely that the replacement of the hydrophobic leucine residue with a small polar serine residue in cIFNAR1 disrupts hydrophobic contacts to induce a conformational change of the loop in cIFNAR1. Since the loop harbors the ligand-contacting helix (Fig. 3a), the L262S substitution in cIFNAR1 would perturb proper interactions of cIFNAR1C with the ligand. The importance of propping a loop engaged in protein–protein interactions was presented in a previous study where the substitutions of a tyrosine residue in TNFα with a small amino acid reduced the receptor binding affinity (Cha et al. 1998).

The structural effect of the cetacean-specific E118K replacement observed in cetacean IFNε (cIFNε) was examined using the crystal structure of the human IFNω-IFNAR complex (PDB code: 3SE4). We built up the in silico structure of human IFNε (hIFNε) with the crystal structure of IFNβ (PDB code: 1AU1) as a template, and the template-based model structure of hIFNε was then superposed onto that of IFNω in the IFNω-IFNAR complex. Notably, the negatively charged side chain of Glu118 forms a salt bridge with the positively charged guanidium side chain of Arg268 in hIFNAR1 (Fig. 3b). Since the arginine residue in cIFNAR1 is conserved, the E118K mutation in cIFNε is highly likely to provoke charge repulsion with Arg268 in cIFNAR1, which should exert a negative effect on the receptor binding.

STAT1 is a major component in the IFN-mediated signaling pathway (Platanias 2005), and treatment of recombinant IFNε with RAW264.7 cells was known to phosphorylate Y701 of STAT1 (Stifter et al. 2018). Cetacean STAT1 was found to have three specific amino acids compared with terrestrial mammals (Table S6). Two (620S > P and 626P > S) of these three amino acids were included in the Src homology 2 domains (SH2) and were predicted to cause functional change.

## Discussion

We investigated the genetic adaptation by which marine mammals cope with abundant ocean viruses via evolutionary analysis of cetacean-specific virus-responsive genes. We found significant signatures of evolution in 21 genes and potentially function-altering mutation in the 58 genes present in ancestral cetaceans. Cetaceans inhabit the oceans and seas in which viruses are abundant, and cetacean mucosa in the nasal cavity, oral cavity, respiratory system, gastrointestinal tract, and reproductive tract could be more frequently exposed to external stimulus than terrestrial mammals. And innate immunity in mucosa represents the body’s first line of defense against myriad of stresses (McGhee and Fujihashi 2012). However, cetacean-specific mucosal immune systems have not been investigated at the genome scale. The identification of evolutionary marks in cetacean genes related to mucosal immunity suggest that cetacean mucosa may be equipped with immune systems adjusted to virus-rich marine environments.

Cytokines are small proteins that regulate immunity, inflammation, and hematopoiesis and thus play important roles in a number of physiological and pathophysiological functions, including mucosal immunity (Khan 2008). Interestingly, many cytokines detected as genes with cetacean-specific function-altering mutations or positive selected genes such as IFN-ε, IL-6, and IL-27 are implicated in mucosal immunity.

IFN-ε belongs to the type-I IFN family and has unique expression and functional properties. The distinct role of IFN-ε in antiviral activity is related to mucosal immune regulation (Day et al. 2008). Type I IFNs act in complex with the IFN-α/β receptor (IFNAR) composed of IFNAR1 and IFNAR2. IFN-ε shows a high affinity for IFNAR1 (Stifter et al. 2018), which we also identified as PSG in our analysis. As shown in Fig. 3, the cetacean-specific substitutions are predicted to weaken the interaction between the ligand and its receptor, which generally attenuate biological activity, including immune response. Another function of mucosa immunity is to tone down immune responses to pathogens encountered on a daily basis, which is called immune tolerance. Cetacean mucosae repeatedly contact with tons of aquatic antigens, and marine mammals may suffer from hyperimmune response if they do not properly manage the response. Interestingly, recent genome analysis on pangolins, which are covered with thick and hard scales, showed that the single copy gene *IFNE* is pseudogenized (Choo et al. 2016). The evolution in cetacean IFN-ε and IFNAR1 raised two possibilities that cetacean mucosa evolved to reinforce the defense against viruses or to alleviate the hypersensitivity to ocean antigens. These findings suggest that the effects of cetacean-specific replacements in IFN-ε and IFNAR1 on mucosal immunity should be further explored.

Cytokine *IL-6* is a gene that contains function-altering mutations among cetacean virus-responsive genes. *IL-6*-deficient mice failed to properly respond to viral infection (Kopf et al. 1994), and the number of immunoglobulin A (IgA)-producing cells at their mucosae was greatly reduced (Ramsay et al. 1994), which demonstrated that IL-6 has an effect on mucosal immunity. It has also been reported that IL-6 upregulated *IFN-ε* expression depending on cell type, cytokine concentration, and incubation time (Couret et al. 2017), which suggests that IL-6 and IFN-ε could cooperatively support cetacean mucosal immunity. IL-27 is known to play an important role in protecting against excessive inflammation during influenza virus infection (Liu et al. 2014) and functions as a critical immunoregulatory cytokine, especially for T cells (Bosmann and Ward 2013). Therefore, IL-27 has been investigated to treat the diseases caused by dysfunctions of immune systems in mucosa, such as nasal allergies (Suzuki et al. 2019) and Inflammatory Bowel Disease (Andrews et al. 2016). Treatment with IL-27 before the herpes simplex virus-1 infection also prevented the virus release by secretion of IL-6 and other chemokines (Heikkilä et al. 2016). On the other hand, upon respiratory viral infection, the IL-6 functions as a critical anti-inflammatory regulator of immunopathology via induction of IL-27 (Pyle et al. 2017). Taken together, IL-6 and IL-27 may exert a combinatorial effect on cetacean defense systems against viruses.

JAK (Janus kinase)-STAT is the main signaling pathway activated by IFNs and is associated with killing viruses within infected cells by upregulating hundreds of interferon-stimulated genes (ISGs) (Platanias 2005). Moreover, IFN-ε has a preference for IFNAR1 rather than IFNAR2 and induces STAT1 phosphorylation (Stifter et al. 2018). In addition to IFN-ε and IFNAR1, cetacean STAT1 also contains uAAS predicted to be functional alteration. Therefore, STAT1 may be involved in cetacean-specific antiviral systems.

Epidemics and sporadic disease caused by cetacean morbilliviruses have been observed in different cetacean species and lots of symptom and dysfunction have been reported. However, defense mechanism against morbilliviruses was not investigated (Beineke et al. 2010). Toll-like receptors (TLRs) are a family of proteins that play an important role to protect from pathogen and cetacean TLR was considered to be a good candidate to study adaptive evolution of marine mammals (Shen et al. 2012). Several studies reported positive selection in cetacean TLR suggesting adaptive evolution in cetacean innate immunity (Ishengoma and Agaba 2017; Tian et al. 2019; Xu et al. 2019). Interestingly, Myxovirus (Mx1) and Myxovirus 2 (Mx2) antiviral proteins are nonfunctional in odontocetes, toothed whales but not in mysticetes, and baleen whales (Braun et al. 2015). These proteins inhibit DNA and RNA viruses by blocking of viral replication cycle (Haller et al. 2015). While the research of cetacean defense system against environmental insult has dealt with a candidate genes or protein family so far, our analysis focused on a group of cetacean virus-responsive genes.

**Fig. 4:**
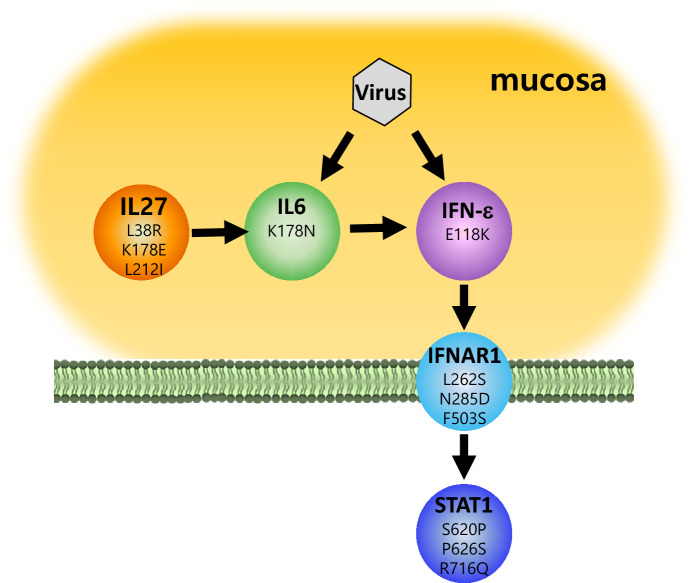
Summary of cetacean virus-responsive genes involved in mucous immunity. The numbers in circles indicate cetacean-specific function-altering amino acid replacement (Table S6)

In conclusion, our evolutionary analysis on virus-responsive genes of cetaceans identified 11 REGs and 10 PSGs and identified 58 cetacean uAASs, some of which are related to mucosal immunity and type I IFN signaling. Since the mucosal immune response of cetaceans is assumed to be necessary for adaptation in aquatic environments, genes with cetacean-specific mutations such as *IFN-ε*, *IL-6*, *IL-27*, and *IFNAR1* provide insight about how cetaceans deal with ocean pathogens, including viruses (Fig. 4). However, the cetacean-specific amino acids in virus-responsive genes may be not definite since the genomes in this study did not cover all mammals. The results will be confirmed by adding more mammalian genomes later, including other marine and terrestrial mammals.

## Supplementary Information

Below is the link to the electronic supplementary material.

Supplementary file1 (XLSX 88 KB)
